# Closed Gastroschisis with Vanished Small Bowel and Jejunal Atresia

**DOI:** 10.21699/jns.v5i4.416

**Published:** 2016-10-10

**Authors:** RS Sisodiya, SS Panda, CK Gupta, SK Sinha

**Affiliations:** Department of Pediatric Surgery, Maulana Azad Medical College and Associated LNH, New Delhi -110002, India

**Dear Sir**

Closed gastroschisis is a congenital abdominal wall defect where the defect has been closed around the viscera and manifest in various clinical ways. [1,2] We present a case of closed gastroschisis with short bowel and intestinal atresia.

Pre-term (35 weeks) 1-day old (2.1kg) male child delivered by Caesarean section (in view of meconium stained liquor) presented with a fleshy red mass protruding from right side of umbilical cord since birth. Examination revealed soft mass (mummified mass of bowel) attached with abdominal wall just right to umbilical cord (Fig.1). Laboratory investigations were normal. Abdominal X-ray gave impression of proximal small bowel atresia and extra abdominal soft tissue mass with no calcification in it. Intra-operatively there was tiny defect in muscle just right to umbilicus. Matted (mummified) vanished small bowel was connected with cord like structure to atretic end of jejunum proximally and transverse colon distally (entry-exit atresia) without any lumen. After resection of the matted bowel, only 25cm of jejunum and micro transverse colon were left. Bishop Koop type stoma (jejuno-transverse) was fashioned. A central line (right internal jugular vein) was inserted. Post operatively child remained on total parental nutrition. Oral pre-digested feed was started on 9th post-operative day but child developed complications of short bowel syndrome like watery diarrhoea, electrolyte imbalance, weight loss and central line related sepsis. Although we have closed Bishop Koop stoma 3 weeks after first surgery but ultimately child succumbed within 4 weeks of admission.

**Figure:**
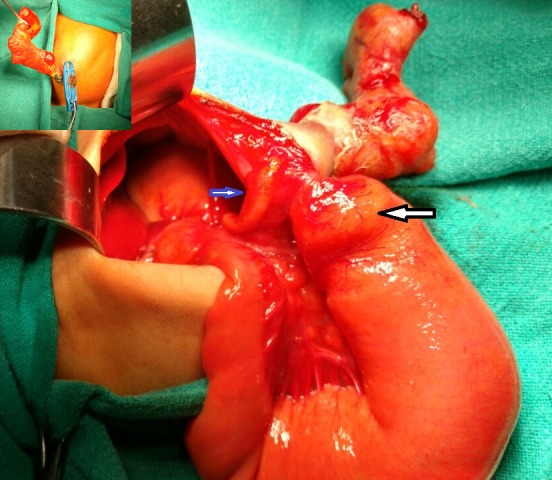
Figure 1: Showing dilated jejunum with atresia (big arrow) and distal transverse colon atresia (small arrow). Inset shows matted bowel mass exiting from a tiny hole in the muscle just right to the umbilical cord.

Incidence of closed/closing gastroschisis is 6% of all gastroschisis.[1] There are various possible phenotypic presentation of closing gastroschisis, at one extreme end, there is closed abdominal ring with viable viscera; at other extreme it would be intestinal atresia at abdominal ring or infarction of midgut, intestinal resorption (matted thick fibrotic) and normal appearing abdominal wall termed as vanishing midgut.[1-4] 

Embryological event leading to closed gastroschisis and its various phenotypic presentation is still not clear.[1-4] The plausible explanation could be that this closing of abdominal ring cause narrowing of exiting bowel and subsequently entering bowel that leads to ischemia of bowel and subsequent various phenotypic presentation like atresia, midgut gangrene, bowel resoption.[1,4,5]

In one study, vanishing bowel was classified phenotypically as Type I: vanishing gut with Lumen, Type II: vanishing gut without lumen or nubbin of tissue, Type III: antenatal evidence of gastroschisis and at birth total absence of midgut.[4] This case exemplify type II vanishing bowel. Infants born with gastroschisis and vanishing midgut have nearly 70%-75% mortality.[2] Mortality is due to short bowel syndrome, central line related sepsis and in long run total parental nutrition related liver disorder.[1,2,5]

## Footnotes

**Source of Support:** Nil

**Conflict of Interest:** None
